# All-trans-retinoic acid suppresses rat embryo hindlimb bud mesenchymal chondrogenesis by modulating HoxD9 expression

**DOI:** 10.1080/21655979.2021.1940613

**Published:** 2021-07-21

**Authors:** Quan Hong, Xue-Dong Li, Peng Xie, Shi-Xin Du

**Affiliations:** a Department of Orthopedics, Jieyang People’s Hospital (Jieyang Affiliated Hospital, Sun Yat-sen University), Jieyang, Guangdong, China; b Department of Orthopedics, Shenzhen Luohu Hospital Group Luohu People’s Hospital (The Third Affiliated Hospital of Shenzhen University), Shenzhen, Guangdong, China

**Keywords:** All-trans retinoic acid, club foot, chondrogenesis, HoxD9

## Abstract

In vertebrates, 5ʹ-Hoxd genes (Hoxd9), which are expressed in the hindlimb bud mesenchyme, participate in limb growth and patterning in early embryonic development. In the present study, We investigated the mechanisms by which ATRA regulates cultured E12.5 rat embryo hindlimb bud mesenchymal cells (rEHBMCs). Following exposure to ATRA over 24 h, mRNA and protein expression levels of HoxD9 were evaluated by reverse transcription-polymerase chain reaction (RT-PCR), quantitative real-time PCR (qPCR), and western blotting. Flow cytometry was used to detect apoptosis. ATRA inhibited the condensation and proliferation, and promoted the apoptosis rate of the rEHBMCs in a dose-dependent manner. Sox9 and Col2a1 in rEHBMCs were downregulated by ATRA in a dose-dependent manner at both mRNA and protein levels. Similarly, HoxD9 was downregulated by ATRA in a dose-dependent manner, in parallel with the cartilage-specific molecules Sox9 and Col2a1. Both qPCR and western blotting showed that both Shh and Gli3 were downregulated. Overexpression of HoxD9 reversed the effects of ATRA. These results demonstrate that ATRA suppresses chondrogenesis in rEHBMCs by inhibiting the expression of HoxD9 and its downstream protein targets, including Sox9 and Col2a1. This effect may also be correlated with inhibition of the Shh-Gli3 signaling pathway.

## Graphical abstract

**Figure:**
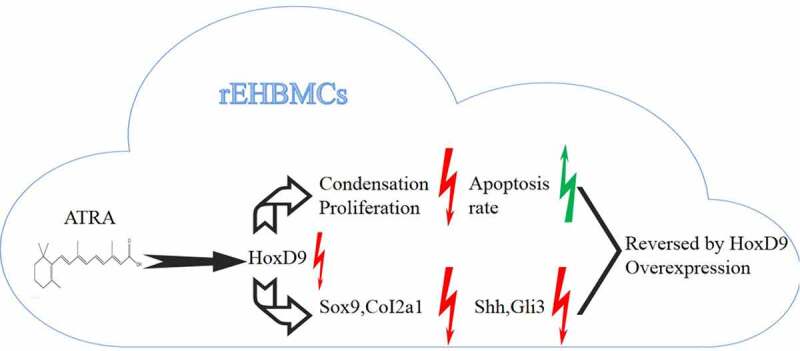

## Introduction

Congenital clubfoot (CCF), or talipes equinovarus, is one of the most common congenital malformations that seriously compromises child health, occurring in approximately one of every thousand live births [1,2]. Because clubfoot is a heterogeneous, multifactorial disorder, its exact etiology has not yet been fully elucidated [3]. At present, it is believed that, the occurrence of CCF is the result of genetic and environmental factors. Recent research reports showed that the pathogenesis of CCF is closely related to the changes of several related genes [4,5].

The retinoic acid (RA) signaling pathway plays an important role in limb development [6]. Vertebrate limb growth and patterning depend on two distinct and independent, yet coordinated systems for two axes: one for anterior-to-posterior another for proximal-to-distal [7]. Hox genes have been implicated in vertebrate limb development of mice and chicks via gene expression analyses [8,9]. Substantial evidence has shown that Hox genes are essential for limb development, where they participate in the growth and organization of body structures [10]. Within the Hox gene family, the regulation and function of the HoxA and HoxD clusters have been largely documented by gain-of-function and loss-of-function mutations in mammals. The complete absence of HoxA and HoxD leads to heavily truncated limbs and only a proximal remnant of the humerus [11]. However, deletions of HoxB and HoxC clusters do not induce limb abnormalities [12]. Thus, it is suggested that both HoxA and HoxD play indispensable roles in vertebrate limb development, since their deletions contribute to congenital malformation [13]. The zone of polarizing activity (ZPA) controls distal limb patterning, including the number and identity of digits, and is mediated by sonic hedgehog (Shh) [14]. Shh signaling is mediated by three Gli genes in vertebrates (Gli1, Gli2, and Gli3). Of these, Gli3 is essential for limb development, as evidenced by the polydactylous phenotype of the extra-toes (*Xt*) Gli3 mutant allele [15]. Interestingly, it has been recently shown that Gli3 and HoxD12 interact both genetically and physically to modulate the function of Gli3R. This suggests that HoxD proteins may function semi-quantitatively to regulate digit patterns and identities by interacting with Gli3 [16].

All-trans-retinoic acid (ATRA) belongs to the vitamin A family, which can up-regulate the expression of osteogenic related genes and promote matrix mineralization. However, the previous study also showed that ATRA can promote the proliferation and differentiation of osteoclasts [17]. Under the action of low dose ATRA, osteoblasts can further proliferate and promote the growth of bone and cartilage. It has been reported that appropriate ATRA can promote the differentiation of rat adipose stem cells into osteogenic cells without activating osteoclasts [18]. On the other hand, ATRA also plays a positive role in inhibiting cell proliferation and differentiation. Under the action of high dose of ATRA, growth and morphogenesis of the bone will be strongly inhibited, and the bone resorption function of osteoclasts will be stimulated [19]. In our previous studies, we used all-trans-retinoic acid (ATRA) to establish a CCF-like model *in vivo* by inhibiting the development of hindlimb cartilage [20]. This model was used to investigate the effects of ATRA on the chondrogenesis of rat embryo hindlimb bud mesenchymal cells (rEHBMCs) and their specific underlying cellular mechanisms.

Therefore, this study aimed to explore the effect of ATRA on the chondrogenesis of rEHBMCs through establishing a CCF-like model in vitro. We hypothesized that ATRA exerts its roles via inhibiting Hoxd9 levels and the Shh-Gli3 signaling pathway.

## Materials and methods

### Animals and cell culture

Sprague-Dawley (SD) rats weighing 240–280 g were supplied by the Animal Center, Shantou University Medical College (Shantou, China). This study was carried out in strict accordance with the recommendations outlined in the ‘Guide for the Care and Use of Laboratory Animals’ by the National Institutes of Health. The protocol was approved by the Animal Management Rules of China (Permit Number 55–2001, Ministry of Health, China) and the Guide for Care and Use of Laboratory Animals, Shantou University Medical College. All surgeries were performed under sodium pentobarbital anesthesia and all efforts were made to minimize suffering. All animals were housed in a controlled environment (22°C and a 12 h light/dark cycle, with a light period from 6:00 to 18:00) with *ad libitum* access to standard laboratory chow and water. Rats were mated overnight. The date when a vaginal plug was first detected was considered as day 0 of pregnancy. On day 12.5 of pregnancy, female rats received intraperitoneal anesthesia, then were euthanized by decapitation and immersion in 75% alcohol for 5 min.

The hindlimbs of the fetuses were excised under sterile conditions. Briefly, the uterus was dissected from the abdomen and washed three times with phosphate buffer solution (PBS). After this, the uterus was transferred to a glass culture dish, where the fetus and outer membrane were separated from the uterus using ophthalmic scissors. The amnion, chorion, vitelline, and chorioallantoic membranes were carefully removed; then the hindlimbs of the fetuses were cut off and collected in a centrifuge tube. After tryptic digestion, rEHBMCs were diluted in Dulbecco’s modified Eagle’s medium (DMEM) (Sigma, St. Louis, MO, USA) with 10% (v/v) fetal bovine serum (FBS) (Hyclone, Logan, UT, USA) and 1% penicillin/streptomycin (v/v) (Invitrogen, Carlsbad, CA, USA). This was then plated in 60 mm plates at a density of 1 × 10^6^ cells per well and incubated at 37°C for 12 h in a humidified atmosphere of 95% O_2_ and 5% CO_2_. Thereafter, the medium was replaced with DMEM containing 1% (v/v) FBS and 1% penicillin/streptomycin (v/v), and incubation was continued for an additional 12 h. ATRA (Sigma, St. Louis, MO, USA) was dissolved in DMSO (Sigma, St. Louis, MO, USA) and added to cultures in a dilution series (0.01, 0.1, 1, and 10 µmol/L), maintaining DMSO levels below 0.05% (v/v). DMSO alone was added to control cultures at a final concentration of 0.05% (v/v). The concentrations selected were based on a previous study [21].

### Alcian blue staining

Cells were plated in fibronectin-coated 6-well plates at a density of 1 × l0^5^ cells/well and incubated for 24 h. After treatment with ATRA at concentrations of 0.01, 0.1, 1, and 10 µmol/L for another 24 h, proteoglycan expression in rEHBMCs was determined by Alcian blue staining . Briefly, cells were washed three times with double-distilled water, fixed in 4% (w/v) paraformaldehyde (Sigma, St. Louis, MO, USA) for 30 min, and then stained with neutral red for 15 min. Cells were then rinsed three times with double-distilled water and stained with 1% (w/v) Alcian blue (Boster Co., Ltd., Wuhan, China) for 30 min. Thereafter, the cells were dehydrated using 95% ethanol. Samples were observed using a Nikon TE2000-S inverted microscope (Nikon, Japan), and images were imported into Image-Pro Plus software (version 6.0; Media Cybernetics, Bethesda, MD, USA) for quantification. The area of blue staining was counted as the quantitative result.

### Cell counting kit-8 assays

The cell viability was measured as previous study described [22]. Cells were plated in fibronectin-coated 6-well plates at a density of 1 × l0^5^ cells/well and incubated for 24 h. After treatment with ATRA at concentrations of 0.01, 0.1, 1, and 10 µmol/L for another 24 h, the proliferation of rEHBMC cells was assessed at 0, 6, 12, 24, and 48 h using a CCK-8 kit (Beyotime, Shanghai, China) according to the manufacturer’s instructions. The optical density (OD) value at 450 nm was detected using a microplate reader (Bio-Rad, CA, USA).

### Flow cytometry

The cell apoptosis was detected accroding to a previous study [23]. Cells were plated in fibronectin-coated 6-well plates at 1 × l0^5^ per well and incubated for 24 h, then treated with ATRA at the concentrations indicated above. After 24 h, the cells were washed with ice-cold PBS, fixed in ethanol for at least 1 h at – 20°C, washed and stained with propidium iodide (30 g/ml) and Annexin V-FITC, and then treated with 0.6 mg/ml RNase in PBS plus 0.5% (v/v) Tween 20 and 2% FBS. Stained cells were analyzed with a FACSCalibur flow cytometer (BD Bioscience, Franklin Lakes, NJ, USA) using CellQuest software. Approximately 10,000 cells were counted for each sample. Data were analyzed using WinMDI software (version 2.9, Bio-Soft Net) to quantify apoptosis.

### Terminal deoxynucleotide transferase dUTP nick end labeling (TUNEL) assay

This assay was performed using a detection kit (Roche, Mannheim, Germany) in accordance with the manufacturer’s instructions. Green dots indicate apoptotic positive cells and were used to identify TUNEL-positive nuclei. The percentage of apoptotic cells to total cardiomyocytes, indicated by DAPI-positive staining, was also calculated.

### Reverse transcription-polymerase chain reaction (RT-PCR)

Total cellular RNA was isolated using TRIzol reagent (Invitrogen, MA, USA) according to the manufacturer’s instructions. Reverse transcription was performed using a commercially available kit as instructed by the manufacturer. First-strand cDNA synthesis was performed using a Quantscript RT kit (Tiangen Biotech, Beijing, China) from 1 g of total RNA. PCR was performed using a thermocycler (Bio-Rad, Hercules, CA, USA) at 94°C for 30 s, 60°C for 30 s, and 72°C for 60 s, for a total of 30 cycles. Assays were performed for the following genes: Sox9 (XM_001081628), Col2a1 (NM_012929), GAPDH (NM_017008), HoxD9 (NM_001173469), HoxD10 (NM_001107094), HoxD12 (NM_001191903), HoxD13 (NM_001105886), Shh (NM_017221), and Gli3 (NM_080405). Primer sequences are listed in Table 1. Gene expression was normalized to that of GAPDH. PCR products were resolved by agarose gel electrophoresis and imaged under a UV transilluminator after staining with GoldView. Densitometric analysis was performed using Quantity One software version 4.5.2 (Bio-Rad, Hercules, CA, USA). For real-time PCR, reactions were performed using the SYBR PrimeScript RT-PCR kit (Takara Bio, Dalian, China) according to the manufacturer’s instructions in an ABI 7500 system (Applied Biosystems, CA, USA).

**Table 1.** Sequences for real-time PCR primers

Gene	Accession number.	Sequences (5ʹ–3ʹ)	Product length (bp)
Sox9	XM_001081628	F:AGTACCCGCATCTGCACAAC	88
		R: ACGAAGGGTCTCTTCTCGCT	
Col2a1	NM_012929	F:TGGACGATCAGGCGAAACC	244
		R: GCTGCGGATGCTCTCAATCT	
Hoxd9	NM_001173469	F: GGACTCGCTTATAGGCCATGA	141
		R: GCAAAACTACACGAGGCGAA	
Hoxd10	NM_001107094	F:GACATGGGGACCTATGGAATGC	129
		R: CGGATCTGTCCAACTGTCTACT	
Hoxd12	NM_001191903	F: CAGTCGCCAGACTCTTTCTACT	235
		R: GCTCTTCGGGTCCGTCTTT	
Hoxd13	NM_001105886	F: CTTCGGCAACGGCTACTACAG	124
		R: TGACACGTCCATGTACTTCTCC	
Shh	NM_017221	F: CTCGCTGCTGGTATGCTCG	176
		R: ATCGCTCGGAGTTTCTGGAGA	
Gli3	NM_080405	F: GAAGTGCTCCACTCGAACAGA	125
		R: GTGGCTGCATAGTGATTGCG	
GAPDH	NM_017008	F: TGTGGGCATCAATGGATTTGG	116
		R: ACACCATGTATTCCGGGTCAAT	

### Enzyme-linked immunosorbent assay (ELISA)

Col2a1 content in the cell culture supernatant was determined using a commercially available kit (Biosynthesis Biotechnology Co., Beijing, China) according to the manufacturer’s instructions. Absorbance was read using a microplate reader (Model 550 Microplate Reader; Bio-Rad) at 450 nm. The lower and upper limits of detection were 1 and 22 ng/ml, respectively.

### Western blotting

Cell lysates were prepared using conventional methods. SDS-PAGE and blotting were performed as a previously study described [24]. Primary antibodies against the following proteins were used: Sox9 and HoxD9 (both from Abcam, Cambridge, UK), as well as Shh, Gli3, and GAPDH (all from Proteintech, Chicago, IL, USA). Protein bands were visualized using a SuperSignal western blotting Detection Kit (Pierce Biotech. Co., Rockford, IL, USA). Densitometric analysis was performed using Quantity One software (version 4.5.2; Bio-Rad). Protein expression values were normalized to those of GAPDH.

### Statistical analysis

Data are presented as means ± standard deviations. Comparisons between groups were analyzed by one-way ANOVA followed by the *post hoc* Student-Newman-Keuls test (SPSS 17.0 Software). Statistical significance was set at *P* < 0.05.

## Results

In present study, we aimed to explore the effect of ATRA on the chondrogenesis of rEHBMCs through establishing a CCF-like model in vitro. We hypothesized that ATRA suppresses the condensation and proliferation, and promotes the apoptosis rate of the rEHBMCs via inhibiting Hoxd9 levels and the Shh-Gli3 signaling pathway.

### ATRA inhibits rEHBMC condensation and proliferation

**Figure 1.**
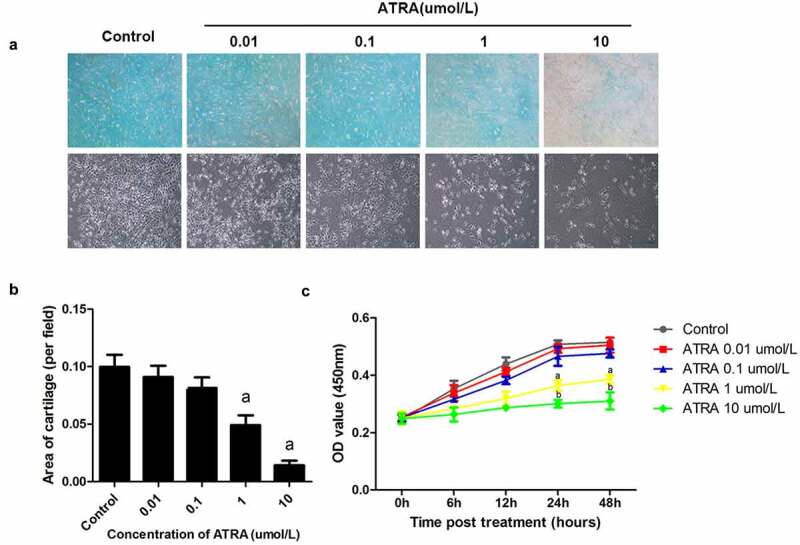
**ATRA suppresses chondrogenesis of rEHBMCs**. (a) and (b) rEHBMCs were treated with ATRA at the indicated concentrations and stained with Alcian blue following 24 h of culture (magnification, x100). Quantification of chondrogenesis was determined by measuring the area of blue staining. (c) Cell proliferation was examined at 0–48 h by CCK-8 assay after treatment of rEHBMCs with ATRA as indicated. Data are shown as the means ± SD of at least three independent experiments. *^a^P <* 0.05 vs control group

To determine whether ATRA affected chondrogenesis, we examined the effects ATRA had on the precartilage condensation of rEHBMCs *in vitro* by using Alcian blue staining, the results showed that the expression of acidic mucopolysaccharide in ATRA treated group was significantly decreased in a dose dependent manner (Figure 1(a)). Then calculated the area of cartilage nodules . ATRA reduced the area of cartilage nodules in a dose-dependent manner, with concentrations of 0.01 and 0.1 µmol/L causing no obvious differences in the area of cartilage nodules compared to controls, while 1 µmol/L caused a 65.01% reduction and 10 µmol/L resulted in a 90.08% reduction (*P* < 0.05 and *P* < 0.01, respectively) (Figure 1(b)).

We further investigated the effects of ATRA on rEHBMc proliferation (Figure 1(c)). There was no time point at which ATRA at 0.01 and 0.1 µmol/L caused significant differences in proliferation (*P* > 0.05 versus controls). In contrast, ATRA at 1 µmol/L reduced rEHBMC proliferation by 28.35% at 24 h and 25.05% at 48 h, when compared to controls (*P* < 0.05). Moreover, ATRA at 10 µmol/L caused a 40.75% reduction at 24 h and a 39.81% reduction at 48 h (both *P* < 0.05 versus controls).

### ATRA enhances rEHBMC apoptosis

**Figure 2.**
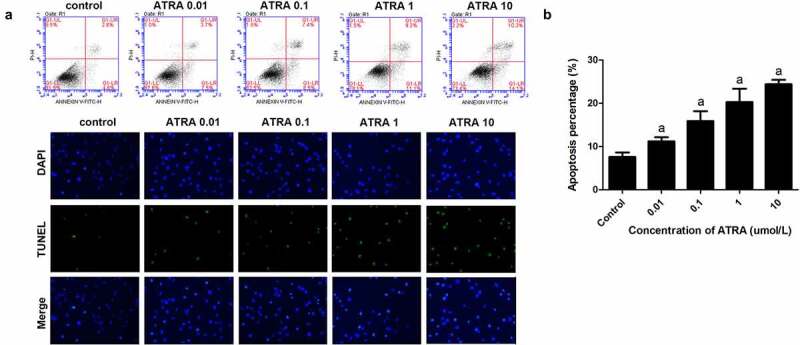
**ATRA enhances apoptotic cell death of rEHBMCs**. (a) and (b) rEHBMCs were treated with ATRA for 24 h at 0.01, 0.1, 1 and 10 µmol/L. Percentages of apoptotic cells were quantified by flow cytometry. (c) Apoptosis was evaluated by TUNEL staining assay. Data are shown as means ± SD of at least three independent experiments. *^a^P <* 0.05 vs control group

Flow cytometry showed that ATRA dose-dependently increased apoptotic cell death from 7.52% in the controls to 23.85% in cells treated with 10 μmol/L ATRA (*P* < 0.01) (Figure 2(a,b)). TUNEL staining also indicated that ATRA promoted the apoptosis of rEHBMCs, the number of positive cells were significantly increased after ATRA treatment in a dose dependent manner (Figure 2(c)). Together these results suggest that ATRA enhances apoptosis of rEHBMCs.

### ATRA dose-dependently reduces the expression of cartilage‑specific molecules in rEHBMCs

**Figure 3.**
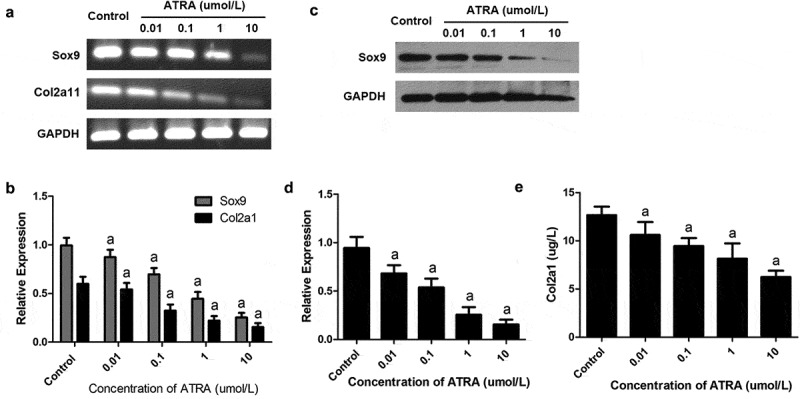
**ATRA inhibits the expression of Sox9 and Col2a1 in rEHBMCs**. rEHBMCs were treated as indicated above. (a) and (b) mRNA levels of Sox9 and Col2a1 were quantified by RT-PCR and qPCR. (c) and (d) Protein levels of Sox9 were determined by immunoblotting. (e) Col2a1 content in the culture supernatant of groups was detected by ELISA. Gene expression was normalized against GAPDH. Data in (b), (d) and (e) are shown as means ± SD of at least three independent experiments. *^a^P* < 0.05 vs control group

Sox9 has been identified as a master transcription factor required for chondrogenesis [25] and, along with Col2a1, has been established as a cartilage-specific marker. Our RT-PCR and qPCR assays revealed that ATRA caused a dose-dependent reduction in the mRNA levels of Sox9C, with 0.1–10 µmol/L ATRA causing a 17.21–61.09% reduction in Sox9 mRNA levels (*P* < 0.01 and *P* < 0.05 versus controls) (Figure 3(a,b)). Consistently, western blotting detected a dose-dependent reduction in the protein expression of Sox9, with all concentrations of ATRA causing marked reductions relative to controls (Figure 3(c,d)) (*P* < 0.01).

We also investigated whether ATRA regulated the expression of Col2a1, the principal constituent of the extracellular cartilage matrix. Using RT-PCR and qPCR assays, we found that ATRA suppressed Col2a1 mRNA levels in a dose-dependent manner. ELISA analyses further revealed an associated dose-dependent reduction in protein content, with ATRA at 10 µmol/L causing a significant decrease in the protein level of Col2a1 (P < 0.01, versus controls) (Figure 3(e)). These results demonstrate that ATRA inhibits the expression of both Sox9 and Col2a1.

### ATRA downregulates the expression of HoxD9 in rEHBMCs

**Figure 4.**
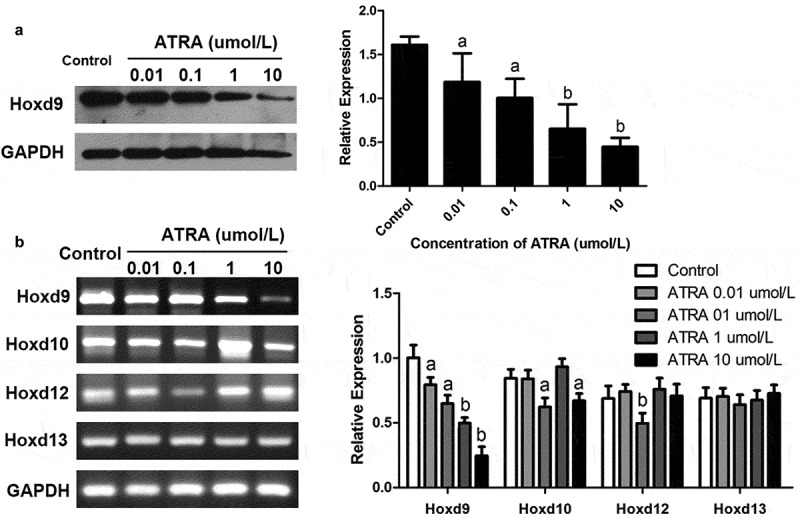
**ATRA inhibits HoxD9 expression in rEHBMCs**. Cells were treated as noted above. (a) Immunoblotting was used to quantify HoxD9 protein expression. Expression levels were normalized to GAPDH. (B) RT-PCR and qPCR assays were performed to quantify the mRNA levels of HoxD9, HoxD10, HoxD12 and HoxD13. Data are shown as means ± SD of at least three independent experiments. *^a^P* < 0.05 vs control group, ^b^*P* < 0.01 vs control group

Given the important role of HoxD in chondrogenesis during early vertebrate embryonic development, we investigated its effects on HoxD expression in rEHBMCs. RT-PCR and qPCR assays showed that ATRA dose-dependently reduced HoxD9 mRNA levels. ATRA at 0.01 and 0.1 µmol/L led to a 10.26–26.61% reduction in HoxD9 mRNA levels (*P* < 0.05), and at 1 and 10 µmol/L ATRA caused a significant difference compared to the controls (*P* < 0.01) (Figure 4(b)). ATRA also reduced the mRNA levels of HoxD10 (*P* < 0.05), except for at 1 µmol/L. For the mRNA levels of HoxD12, only at 0.1 µmol/L did ATRA cause a marked reduction (*P* < 0.01 versus controls). Furthermore, there was no marked difference in the mRNA levels of HoxD13 after ATRA treatment (Figure 4(b)).

We examined the HoxD9 protein levels of rEHBMCs treated with ATRA to test the hypothesis that ATRA inhibits chondrogenesis by modulating the expression of this protein. Immunoblotting assays were consistent with the RT-PCR analysis, demonstrating dose-dependent inhibition with ATRA at 1 and 10 µmol/L (*P* < 0.01) (Figure 4(a)). In summary, these results indicate that ATRA may inhibit chondrogenesis of rEHBMCs by downregulating the expression of HoxD9.

### ATRA suppresses Shh-Gli3 signaling

**Figure 5.**
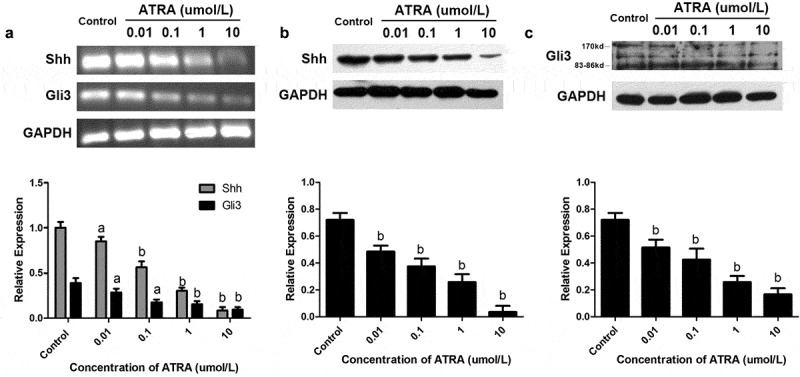
**ATRA suppresses Shh-Gli3 in rEHBMCs**. rEHBMCs were treated with ATRA as noted above. (a) mRNA levels of Shh and Gli3 were quantified by RT-PCR and qPCR assays. Protein expression of Shh (b) and Gli3 (c) was quantified by immunoblotting. Gene expression was normalized to GAPDH. Data are shown as means ± SD of at least three independent experiments. *^a^P* < 0.05 vs control group, ^b^*P* < 0.01 vs control group

Shh is produced by the ZPA during embryonic development and is essential for distal limb skeleton patterning and the specification of digit identities [26]. There is both a disruption in anterior distal development and a loss of posterior identities in Shh-deficient embryos [27]. We were interested in whether ATRA induced changes in the expression of Shh and Gli3 in rEHBMCs. These cells were treated with the concentrations of ATRA indicated above, and RT-PCR and qPCR assays were performed. ATRA dose-dependently reduced the mRNA levels of both Shh and Gli3 (Figure 5(a)), with ATRA at 0.01, 1, and 10 µmol/L causing a significant reduction in the mRNA level of Shh compared to the controls (*P* < 0.01). ATRA at 1 and 10 µmol/L caused a 63.47–82.77% reduction in the expression of Gli3 (*P* < 0.01 versus controls).

Western blotting further demonstrated that ATRA dose-dependently reduced the expression of the Shh protein, with significant reductions in Shh levels occurring at 0.01–10 µmol/L ATRA concentrations (*P* < 0.01 versus controls), suggesting that ATRA inhibits chondrogenesis in rEHBMCs by downregulating Shh protein expression. Furthermore, the protein expression level of Gli3 was detected and a dose-dependent reduction was observed; ATRA at 1 and 10 µmol/L caused a significant reduction compared to the controls (P < 0.01) (Figure 5(b,c)). Therefore, ATRA was found to suppress chondrogenesis in rEHBMCs, which itself demonstrated a positive correlation with the expression of Shh and Gli3.

### Overexpression of HoxD9 reversed the effect of ATRA on rEHBMCs

**Figure 6.**
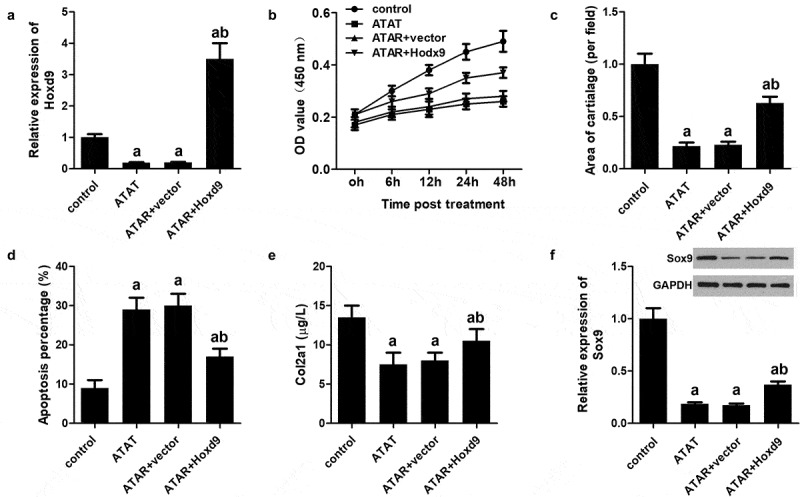
**Overexpression of HoxD9 reverses the effect of ATRA**. rEHBMCs were transfected with the HoxD9 overexpressing vector and the control vector for 24 h and then treated with ATRA for an additional 24 h. (a) qPCR was used to quantify the mRNA expression of HoxD9. (b) CCK-8 was performed to quantify proliferation. (c) Alcian blue staining was used to quantify the chondrogenesis. (d) Flow cytometry was used to quantify apoptosis of rEHBMCs. (e) ELISA was used to quantify Col2a1 expression. (f) Western blotting was performed to quantify the expression of HoxD9. aP < 0.05 vs control, bP < 0.05 vs ATRA + vector

To verify whether HoxD9 exerts its function by modulating the expression of HoxD9, we performed rescue experiments. Our qPCR analysis indicated that HoxD9 overexpression was achieved by transfection (Figure 6(a)). CCK-8 results indicated that HoxD9 reversed the effect of ATRA on the proliferation of rEHBMCs (Figure 6(b)). Moreover, Alcian blue staining and flow cytometry showed that HoxD9 overexpression also reversed the effect ATRA had on the chondrogenesis and apoptosis of rEHBMCs (Figure 6(c,d)). We also further evaluated the expression of Sox9 and Col2a1. The results revealed that HoxD9 promoted the expression of Sox9 and Col2a1, which were initially reduced by ATRA treatment (Figure 6(e,f)).

## Discussion

Investigation of the signaling mechanisms underlying limb development has served as a paradigm for understanding general principles governing embryogenesis [28]. Such investigations have shed light on the etiologies of many limb diseases, particularly inherited ones. Many different signaling molecules converge to generate new tissues during the growth and patterning of the limb [29].

In the present study, we found that ATRA inhibited precartilage condensation and proliferation, while enhancing apoptotic cell death in rEHBMCs. We deduce that apoptosis may be involved in ATRA-retarded chondrogenesis in rEHBMCs. We also found that ATRA reduced the area of cartilage nodules *in vitro*, indicating that it inhibited the precartilage condensation of rEHBMCs. In contrast to the condensation stage of cartilage formation, the differentiation stage is characterized by the expression of specific cellular markers and growth factors.

Sox9 has been identified as the master transcription factor required for chondrogenesis [30] and is expressed in all regions involved in cartilage formation, including regions of mesenchymal cell condensation and differentiation [31]. It regulates expression by binding to the promoters or enhancers of cartilage-specific genes, including Col2a1 [32], aggrecan [33], and cartilage link protein [34]. Col2a1 is the principal constituent of the extracellular cartilage matrix activated by Sox9; it has been well-established as a direct target of Sox9 *in vivo* [35]. These data indicate that Sox9 may be required for cartilage formation and that the Sox9-Col2a1 signaling pathway plays a key role in chondrogenesis. In the present study, we found that ATRA down-regulated the expressions of Sox9 and Col2a1 . These findings suggest that ATRA may retard chondrogenesis by regulating the expression of Sox9 and its downstream target, Col2a1.

The fact that ATRA plays an important role in the rEHBMC chondrogenesis prompted us to further investigate the involvement of other important signaling molecules. The 5ʹ-located HoxD cluster determines skeletal patterning in the limb by regulating the formation and growth of different chondrogenic precursors for skeletal elements. However, the roles of individual cluster members have often been difficult to fully evaluate due to extensive functional overlap between multiple 5ʹHoxD genes [36]. We quantified mRNA levels of 5ʹ-HoxD genes (HoxD9, HoxD10, HoxD12, and HoxD13) in E12.5 rat embryos and showed that ATRA induces a dose-dependent reduction in HoxD9. The decrease in HoxD gene expression paralleled the decrease in both Sox9 and Col2a1 expression. Together, these findings suggest that ATRA retards the chondrogenesis of rEHBMCs by downregulating HoxD9. However, the specific mechanism by which ATRA modulates HoxD9 remains unknown.

In vertebrate embryos, Shh is produced by ZPA and mainly specifies cell identity along the anteroposterior limb bud axis [37]. A lack of Shh results in disruption of distal development and the loss of posterior identities, suggesting that it is essential for patterning the distal limb skeleton and in the specification of digit identities [38]. Substantial evidence implicates HoxD genes in Shh activation in the posterior limb bud mesenchyme [16]. In addition, upregulation and distoanterior expansion of 5ʹ-located HoxD gene expression depends on Shh signaling and results in the establishment of presumptive digit expression domains in the distal limb bud mesenchyme [39]. These findings led us to hypothesize that ATRA modulates the expression of Gli3 by regulating its upstream target, Shh. Interestingly, we found that ATRA dose-dependently reduced mRNA and protein expression of Shh in parallel with a similar reduction in Gli3. However, Shh is not only required very early and transiently for digit patterning, but also continuously during development to ensure sufficient cell numbers to produce the normal complement of digits [40].

## Conclusions

Through our study, we showed that ATRA dose-dependently reduced the expression of HoxD9 in rEHBMCs, which is consistent with the observed Sox9 and Col2a1 expression levels. HoxD9 overexpression reversed the effects ATRA had on rEHBMCs, indicating that ATRA exerts its effects by directly inhibiting HoxD9 levels. Moreover, these effects of ATRA are positively correlated with the expression of Shh and Gli3 in rEHBMCs, suggesting that ATRA suppresses chondrogenesis via the Shh-Gli3 signaling pathway, which remains to be elucidated further.
